# Influence of knee flexion angle on graft bending angle during anterior cruciate ligament reconstruction using the transportal technique

**DOI:** 10.1038/s41598-023-41002-x

**Published:** 2023-08-22

**Authors:** Kwangho Chung, Chong Hyuk Choi, Sung-Hwan Kim, Sung-Jae Kim, Hee Chan Choi, Min Jung

**Affiliations:** 1 The Department of Orthopaedic Surgery, The Arthroscopy and Joint Research Institute, Yonsei University College of Medicine, 134, Shinchon-dong, Seodaemun-gu, C.P.O. Box 8044, Seoul, 120-752 Republic of Korea; 2 The Department of Orthopaedic Surgery, Yonsei University College of Medicine, Seoul, Republic of Korea

**Keywords:** Ligaments, Ligaments

## Abstract

This study aimed to examine change in the graft bending angle (GBA) according to various knee flexion angles in creating femoral tunnel by the transportal technique in ACL reconstruction, and to reveal knee flexion angle minimizing GBA while maintaining stable femoral tunnel characteristics. Patients who underwent ACL reconstruction using the transportal technique between January 2017 and December 2018 were retrospectively reviewed. Patients were classified into three groups according to knee flexion angle when creating femoral tunnel (group 1: < 120° (n = 19); group 2: 120–129° (n = 32); group 3: ≥ 130° (n = 33). GBA was measured on three-dimensional knee model reconstructed from postoperative computed tomography images. The length of the femoral tunnel and posterior wall blow-out were also checked. There was significant difference of GBA between the groups (group 1 = 112.1°; group 2 = 106.4°; group 3 = 101.4°, *p* < 0.001). The knee flexion angle in creating femoral tunnel was negatively correlated with GBA (r = − 0.733, *p* < 0.001). Five patients in group 1 had short femoral tunnel. GBA was influenced by knee flexion angle in creating femoral tunnel and got more acute as the knee flexion angle increased. Considering length and risk of wall blow-out in femoral tunnel, and GBA, knee flexion angle between 120 and 130° could be recommended as appropriate angle to create optimal femoral tunnel in ACL reconstruction using the transportal technique.

## Introduction

The femoral tunnel position in anterior cruciate ligament (ACL) reconstruction has been recognized as an important keystone for successful outcomes. Non-anatomic placement of the femoral tunnel aperture can result in increased instability and graft failure after ACL reconstruction^1,2^. Independent femoral drilling techniques, such as the transportal and outside-in techniques instead of the conventional transtibial technique have evolved to create an intra-articular femoral tunnel aperture on the native femoral footprint^3,4^. However, because a change in the method of creating a femoral tunnel affects the overall characteristics of the femoral tunnel in addition to the placement of the aperture, various factors affected by the change in the tunnel creation method should be considered. One of several changing variables that can have clinical significance is graft bending angle (GBA)^3,5^.

The GBA is defined as the angle between the longitudinal axes of the intraosseous tunnel and intra-articular graft^6^. The abrasive forces could be created at the edge of the tunnel aperture because the direction of the ACL graft changes as it leads to the tunnel inside the bone from within the joint. Accordingly, the acute GBA causing a sharp change in graft path may influence graft healing, graft maturation, and tunnel widening resulting in unsatisfactory results^7–10^. The GBA is significant particularly at the femoral tunnel having a more sharp change of graft path at the tunnel aperture compared to that at the tibial tunnel^8,9^. According to several previous studies on GBAs created by various femoral drilling techniques^5,11,12^, the GBA created by independent femoral drilling techniques including the transportal or outside-in techniques tends to be more acute than the angle created by the transtibial technique^12,13^. When comparing the two methods of the transportal and outside-in techniques, the outside-in technique tends to create more acute GBA^5,11,14^. However, the outside-in technique has room to control the femoral tunnel characteristics including the GBA directly by changing the femoral tunnel exit at the far cortex during tunnel creation^15^. Meanwhile, the femoral tunnel characteristics created by the transportal technique cannot be directly controlled, but can be indirectly determined by the knee flexion angle during femoral drilling^16^. In femoral tunnel drilling using the transportal technique, high knee flexion angle is generally recommended to avoid the posterior wall blow-out and to gain sufficient tunnel length^17,18^. Because GBA is affected by the intraosseous tunnel path, the knee flexion angle during femoral tunnel drilling in the transportal technique influences the GBA at the femoral tunnel. Despite the plausible relationship between the knee flexion angle during femoral tunnel drilling and GBA, to the best of our knowledge, no previous study on this has been conducted to date. Therefore, this study aimed to examine the change in GBA according to various knee flexion angles during femoral tunnel drilling using the transportal technique for ACL reconstruction, and to reveal the knee flexion angle minimizing the GBA while maintaining stable femoral tunnel characteristics. This study was based on the hypothesis that there would be a tendency for change in GBA according to variations in knee flexion angle during femoral tunnel drilling.

## Materials and methods

### Patients

A total of 154 patients who underwent ACL reconstruction performed by a single surgeon between January 2017 and December 2018 were retrospectively reviewed after obtaining approval from the institutional review board of our institution. All methods were performed in accordance with the relevant guidelines and regulations. This study received exemption from informed consent by the Institutional Review Board and research process was performed in accordance with the Declaration of Helsinki. Patients who met the following criteria were included: (1) primary single-bundle ACL reconstruction with the transportal technique using an autogenous quadruple hamstring tendon graft; (2) intra-articular femoral and tibial tunnel apertures created within the footprint of a native ACL based on postoperative CT analysis^19,20^. Exclusion criteria were as follows: (1) revision ACL reconstruction; (2) concomitant ligamentous injuries other than ACL injury; (3) previous operative history of the affected knee; (4) osseous deformity around the knee; (5) lower extremity malalignment (normal mechanical axis line passes 8 ± 7 mm medial to the knee joint center on standing hip-knee-ankle radiographs)^21^; and (6) radiographic osteoarthritis greater than Kellgren-Lawrence Grade 1. After applying the inclusion and exclusion criteria, and 84 patients were included in this study. The patients were classified into three groups according to the knee flexion angle while creating a femoral tunnel with the knee flexed, as much as possible, as previously described^16^. Group one (n = 19), two (n = 32), and three (n = 33) consisted of patients with a knee flexion angle of < 120°, 120–129°, and ≥ 130°, respectively.

### Operative procedure

**Figure 1:**
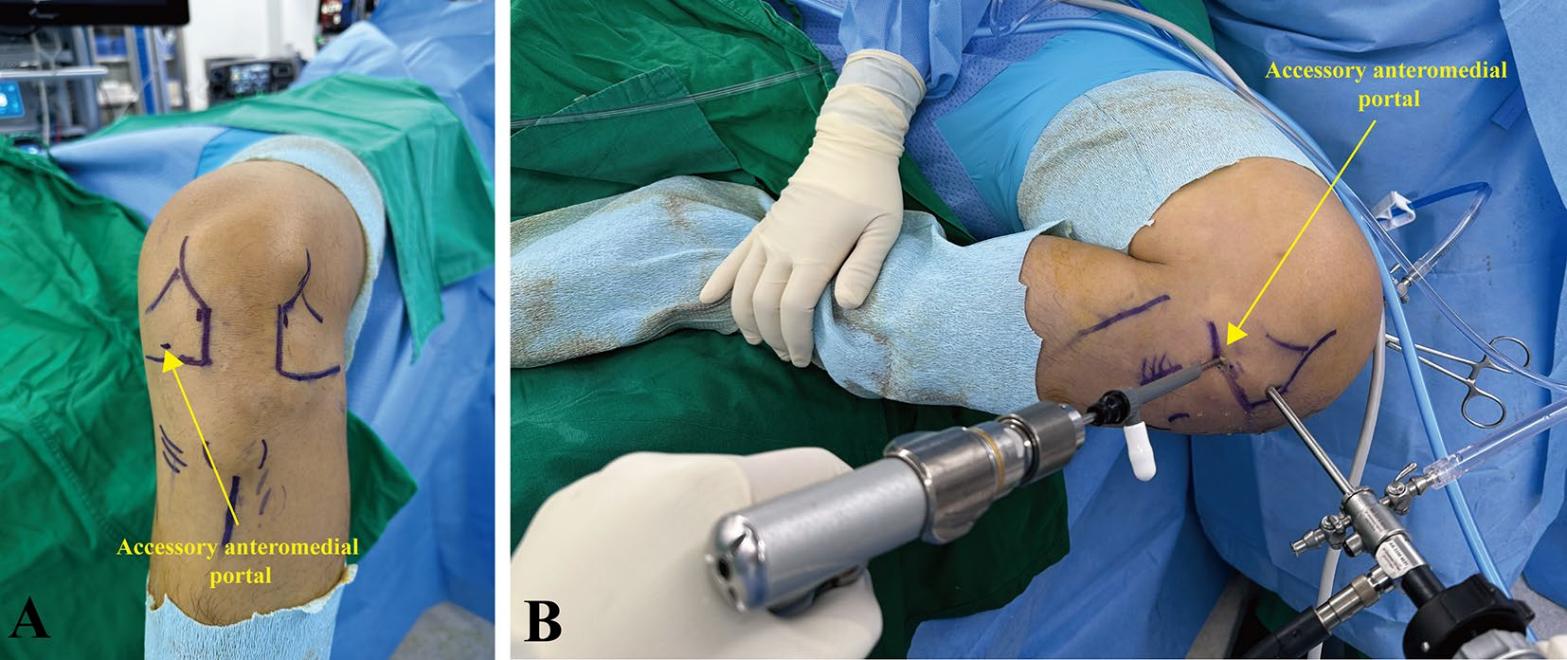
An accessory anteromedial portal placement and the knee position during the femoral tunnel creation. (**A**) An accessory anteromedial portal that could reach the ACL femoral footprint was created just above the medial meniscus, as far away as possible from the medial border of the patellar tendon. (**B**) The knee was flexed as much as possible in a figure-of-four position. The femoral tunnel was created by positioning the rigid mono-fluted reamer closest to the medial femoral condyle cartilage without damaging it. To protect the cartilage of the medial femoral condyle, a plastic cannula was used. A plastic cannula was pushed into the joint to cover the shaft of the reamer located within the joint during drilling.

Arthroscopic ACL reconstruction was performed after harvesting the autogenous quadruple hamstring tendon graft. Apertures of the femoral and tibial tunnels were placed on the footprints of the native ACL with reference to injured ACL remnant tissue and anatomical landmarks^22,23^. The femoral tunnel aperture was created between the lateral intercondylar ridge and the posterior articular margin, referencing the ACL remnant tissue^23^. The center of the tibial tunnel aperture was targeted at about two-fifths of the medial-to-lateral tibial spine width, approximately 15 mm anterior to the PCL, taking account of the ACL remnant tissue^22^. First, a tibial tunnel was created using a tibial guide set to 55°. Thereafter, an accessory anteromedial portal that could reach the ACL femoral footprint was created just above the medial meniscus, as far away as possible from the medial border of the patellar tendon. The knee was flexed as much as possible in a figure-of-four position (Fig. 1)^24^. The Beath pin was passed as close as possible to the medial femoral condyle without cartilage injury through the center of the native ACL femoral footprint from the accessory anteromedial portal. A rigid mono-fluted reamer was carefully inserted over the Beath pin. During insertion of the reamer, the single blade is toward the front of the knee to avoid damaging the cartilage of medial femoral condyle. Afterward, the femoral tunnel was created by reaming the rigid mono-fluted reamer closest to the medial femoral condyle cartilage without damaging it by covering the shaft of the reamer with a plastic cannula. The knee flexion angle during femoral tunnel creation was measured using a sterile goniometer with two 12-inch arms. The greater trochanter, lateral epicondyle of the distal femur, fibular head, and lateral malleolus were used as measurement landmarks based on previous studies^16^. The center of the goniometer was placed on the lateral epicondyle of the femur. Thereafter, the stationary arm of the goniometer was placed parallel to the femoral shaft with reference to the greater trochanter, and the moving arm was placed parallel to the line connecting the fibular head and the lateral malleolus representing the tibial shaft. A single operating surgeon measured and recorded the knee flexion angle. After positioning the graft within the tunnel, the femoral side was fixated with a cortical suspensory fixation device. Afterwards, the tibial side was secured with bioabsorbable interference screw and supplemented with screw-and-washer construct.

### Postoperative computed tomography (CT) three-dimensional (3D) analysis and measurement

A computed tomography (CT) scan was performed on the first day after operation with the knee fully extended. The CT scan was performed using a CT scanner (Sensation 64; Siemens Healthcare, Erlangen, Germany). The scan parameters were as follows: tube voltage, 120 kVp; tube current, 135–253 mA; acquisition matrix, 512 × 512 pixels; scan field of view, 134–271 mm; and slice thickness, 0.6–1 mm. Digital Imaging and Communications in Medicine (DICOM) data of the postoperative CT scan were downloaded from the Picture Archiving and Communication System (Centricity PACS, GE Medical System Information Technologies, USA). The data were then imported into Mimics software (version 17; Materialize, Belgium), and a 3D model of the knee was reconstructed.

**Figure 2:**
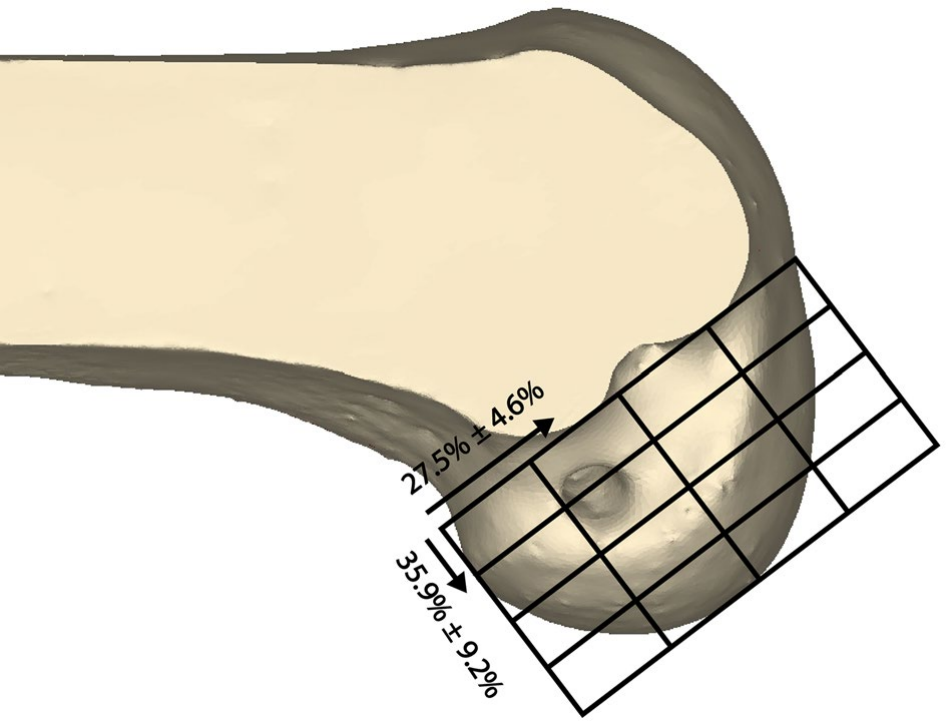
Evaluation of the position of the femoral tunnel aperture by the quadrant method using three-dimensional knee model. The coordinates of the standard area of the ACL femoral footprint center were 27.5% ± 4.6% of the distance parallel to the Blumensaat line measured from the posterior border and 35.9% ± 9.2% of the distance perpendicular to the Blumensaat line measured from the Blumensaat line.

**Figure 3:**
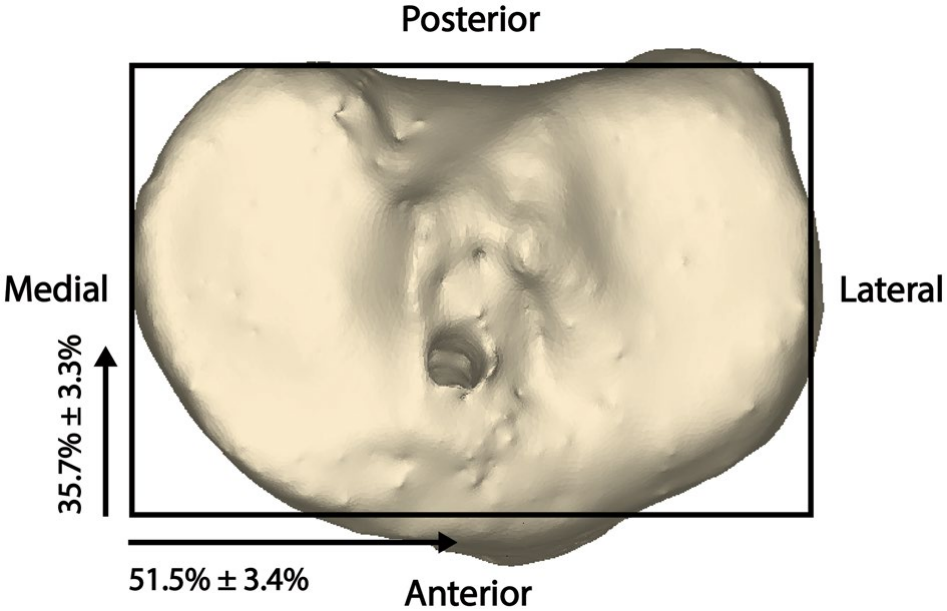
Evaluation of the position of the tibial aperture using axial plane of three-dimensional knee model. The coordinates of the standard area of the ACL tibial footprint center were 35.7% ± 3.3% of the anterior-to-posterior distance and 51.5% ± 3.4% of the medial-to-lateral distance on axial plane.

As mentioned in the inclusion criteria above, as the first process, it was checked whether the intra-articular apertures of the femoral and tibial tunnels were placed in their native ACL footprints^16,17,19,20,25^. The position of the femoral tunnel aperture was evaluated by the quadrant method using a 3D knee model (Fig. 2)^16,26^. A 4 × 4 grid was placed on the medial wall of the lateral femoral condyle from a true medial view of the femur similarly to the quadrant method on the standard lateral radiograph^16^. The Blumensaat line on the lateral radiograph was replaced by the line along the intercondylar roof as a reference for the grid alignment. The coordinates of the standard area of the ACL femoral footprint center were 27.5 ± 4.6% of the distance parallel to the Blumensaat line measured from the posterior border and 35.9 ± 9.2% of the distance perpendicular to the Blumensaat line measured from the Blumensaat line^19^. The position of the tibial tunnel aperture was also evaluated using the coordinates on the axial plane^20,25^. The distances from anterior to posterior and medial to lateral on the axial plane were measured (Fig. 3). The coordinates of the standard area of the ACL tibial footprint center were 35.7 ± 3.3% of the anterior-to-posterior distance and 51.5 ± 3.4% of the medial-to-lateral distance^20,25^. Only cases in which the centers of intra-articular femoral and tibial tunnel apertures were created within the footprint of the native ACL were included in the study.

**Figure 4:**
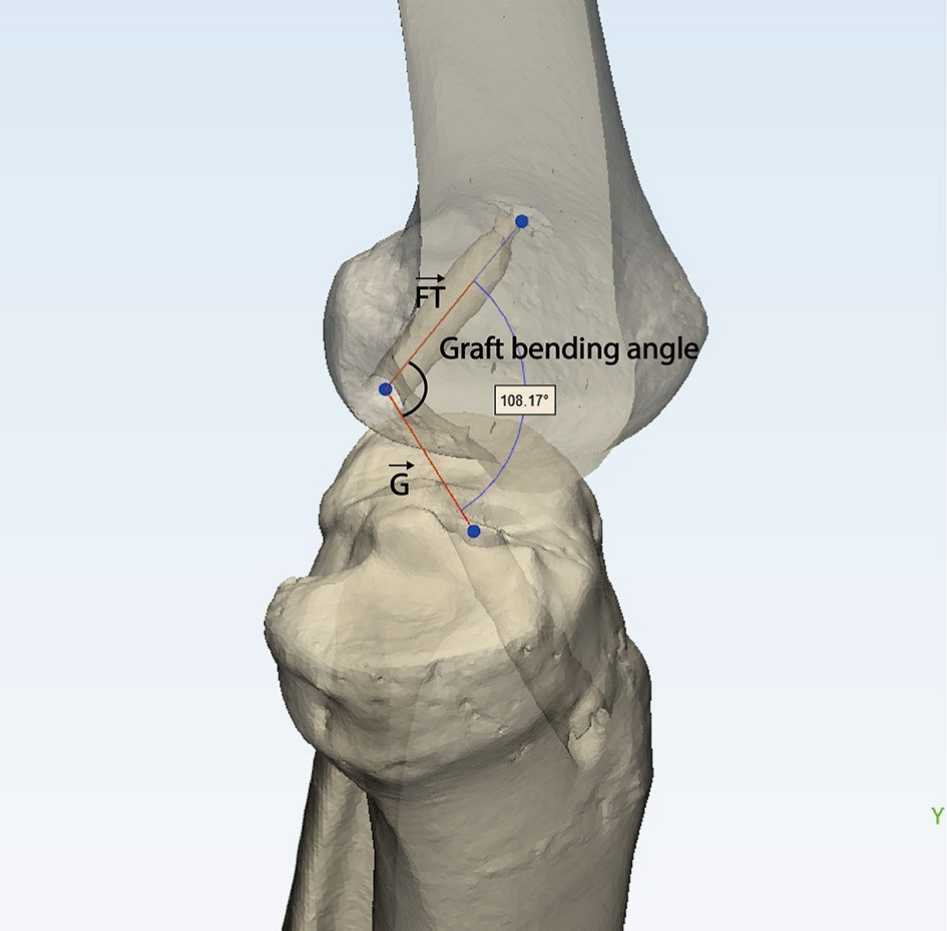
Evaluation of the graft bending angle with three-dimensional knee model. The graft bending angle is formed by the intra-articular graft vector ($\vec{G}$; the vector from the intra-articular femoral tunnel aperture to the intra-articular tibial tunnel aperture) and femoral tunnel vector ($\overrightarrow {FT}$; the vector from the intra-articular femoral tunnel aperture to the femoral tunnel outlet).

The GBA was calculated using the following formula to measure the bending angle in 3D space^9^. The GBA is formed by the intra-articular graft vector ($\vec{G}$; the vector from the intra-articular femoral tunnel aperture to the intra-articular tibial tunnel aperture) and femoral tunnel vector ($\overrightarrow {FT}$; the vector from the intra-articular femoral tunnel aperture to the femoral tunnel outlet) (Fig. 4): ${\text{Graft}}\, {\text{bending}}\, {\text{angle}} \left( \theta \right) = \cos^{ - 1} \frac{{\vec{G} \cdot \overrightarrow {FT} }}{{\left| {\vec{G}} \right|\left| {\overrightarrow {FT} } \right|}}$. According to previous studies^27,28^, stress at the tunnel aperture-graft interface is maximized with the knee in near-full extension. Therefore, GBA was measured using postoperative CT taken in the full extension position. In addition to measuring the GBA, the length of the femoral tunnel and posterior wall blow-out were also checked to identify tunnel characteristics. The femoral tunnel length was measured as the length from the center of the femoral tunnel aperture to the center of the femoral tunnel outlet at the far cortex of the lateral femoral condyle^17^. A femoral tunnel length of 30 mm or more was considered a stable length as described in a previous study^12^. The posterior wall blow-out was defined as the breakage of the posterior wall of the femoral tunnel within the lateral femoral condyle^17^. To increase the reliability, two different orthopaedic surgeons who were blinded to the characteristics of each case measured the graft bending angle and femoral tunnel length. The mean of the two measured numerical values was used.

### Statistical analysis

The Shapiro–Wilk test was used to test for normality. Continuous variables were compared between the groups using analysis of variance (ANOVA). Categorical variables were compared using the Chi-square test or Fisher’s Exact test. For pairwise comparisons between the groups, the Bonferroni correction was employed. Pearson correlation analysis was used to evaluate the relationship between the graft bending angle and knee flexion angle when creating the femoral tunnel. The interobserver reliability of the measurement of variables was evaluated using an intraclass correlation coefficient set as a 95% confidence interval (CI). Significance was set at *p* < 0.05. Statistical analyses were carried out using IBM SPSS Statistics for Windows software program (version 26.0; IBM, USA). The statistical power was calculated using G*Power version 3.1 (Düsseldorf, Nordrhein-Westfalen, Germany)^29^.

### Ethical approval

The study was approved by the Institutional Review Board of Severance Hospital, Yonsei University College of Medicine (4-2022-0377).

### Informed consent

This study received exemption from informed consent by the Institutional Review Board.

## Results

Demographic data are listed in Table 1. There were no differences in sex, age, height, weight, or body mass index between the groups (*p* > 0.05). The mean knee flexion angle when creating femoral tunnel was significantly different between the groups (group one = 116.6 ± 2.3°, group two = 125.7 ± 2.5°, and group three = 133.6 ± 2.8°; *p* < 0.001) (Table 2). The coordinate values of the centers of the intra-articular femoral and tibial tunnel apertures did not differ significantly between the groups (*p* > 0.05). There was a significant difference of GBA between the groups (group one = 112.1 ± 4.1°, group two = 106.4 ± 4.7°, and group three = 101.4 ± 4.6°; *p* < 0.001). The femoral tunnel length also differed between the groups (group one = 32.1 ± 4.1 mm, group two = 36.0 ± 3.8 mm, and group three = 39.6 ± 3.9 mm; *p* < 0.001). The number of patients with a short femoral tunnel of < 30 mm was five in group one, while none were in the other two groups. Posterior wall blow-out was not observed in any of the groups.

**Table 1:** Demographic data for patients.

Variable	Group 1 (n = 19)	Group 2 (n = 32)	Group 3 (n = 33)	*P* value
Sex^a^				
Male	12 (63.2)	21 (65.6)	19 (57.6)	0.793
Female	7 (36.8)	11 (34.4)	14 (42.4)	
Age^b^, years	31.6 ± 8.1	29.3 ± 8.5	29.1 ± 9.2	0.582
Height, cm	170.9 ± 9.0	174.2 ± 7.6	172.4 ± 8.9	0.388
Weight, kg	73.3 ± 13.2	71.6 ± 13.0	70.4 ± 11.6	0.723
Body mass index^b^, kg/m^2^	24.9 ± 2.8	23.5 ± 3.2	23.6 ± 2.7	0.179
Injured side^a^				0.457
Right	13 (68.4)	17 (53.1)	17 (51.5)	
Left	6 (31.6)	15 (46.9)	16 (48.5)	
Elapsed time from injury to surgery	6.8 ± 3.8	6.8 ± 3.8	7.0 ± 2.9	0.954

^a^The values are given as n (%).

^b^The values are given as mean ± standard deviation.

**Table 2:** Comparison of tunnel characteristics between the groups.

Parameter	Group 1 (n = 19)	Group 2 (n = 32)	Group 3 (n = 33)	*P* value
Knee flexion angle when creating femoral tunnel^a^, degree	116.6 ± 2.3	125.7 ± 2.5	133.6 ± 2.8	< 0.001
Coordinate values of the intra-articular femoral tunnel aperture center^a^, %				
The distance parallel to the Blumensaat line	27.5 ± 2.9	27.0 ± 2.6	27.4 ± 2.9	0.757
The distance perpendicular to the Blumensaat line	34.1 ± 5.4	35.7 ± 4.5	35.4 ± 5.4	0.546
Coordinate values of the intra-articular tibial tunnel aperture center^a^, %				
The anterior-to-posterior distance	34.3 ± 1.1	34.7 ± 1.5	34.0 ± 1.0	0.078
The medial-to-lateral distance	50.0 ± 1.3	49.7 ± 1.2	50.3 ± 1.5	0.143
Graft bending angle at 0° of knee flexion angle, degree	112.1 ± 4.1	106.4 ± 4.7	101.4 ± 4.6	< 0.001
Femoral tunnel length^a^, mm	32.1 ± 4.1	36.0 ± 3.8	39.6 ± 3.9	< 0.001
The number of cases with < 30 mm in femoral tunnel length^b^	5 (26.3%)	0	0	< 0.001
Femoral tunnel diameter^a^, mm	7.7 ± 0.7	7.8 ± 0.6	7.4 ± 0.7	0.101
Presence of posterior wall blow-out^b^	0	0	0	–

^a^The values are given as mean ± standard deviation.

^b^The values are given as n (%).

**Figure 5:**
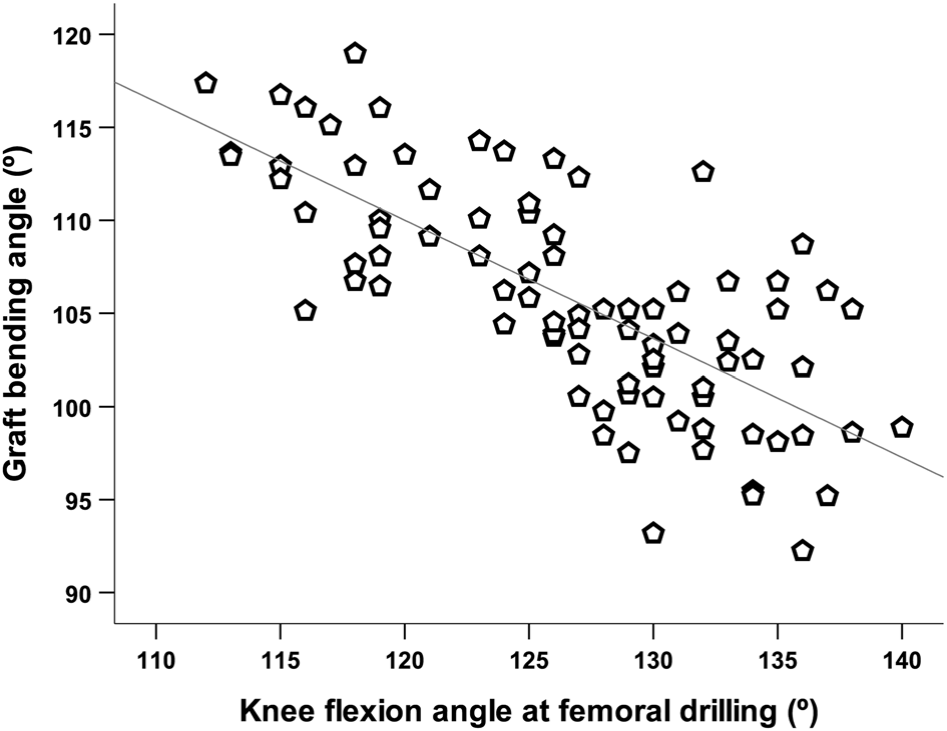
Pearson correlation analysis of graft bending angle and knee flexion angle in creating femoral tunnel. The knee flexion angle was negatively correlated with the graft bending angle measured at 0° of knee flexion (r = − 0.733, *P* < 0.001).

Pearson correlation analysis showed that the knee flexion angle in femoral drilling was negatively correlated with GBA (r = − 0.733, *p* < 0.001) (Fig. 5). The statistical power was calculated to be 1.00 for the comparison of GBA. The intraclass correlation coefficients for interobserver reliability of the tunnel characteristic variables were 0.939 (95% CI 0.747–0.976) for the graft bending angle and 0.914 (95% CI 0.847–0.950) for the femoral tunnel length.

## Discussion

The most important finding of the present study was that the GBA was influenced by the knee flexion angle during femoral tunnel drilling for ACL reconstruction using the transportal technique. Regarding the tendency of the GBA change according to the knee flexion angle during femoral tunnel drilling, the GBA tended to be more acute as the knee flexion angle during femoral tunnel drilling increased. By comprehensively considering the length and risk of wall blow-out in the femoral tunnel, including this effect on the GBA, knee flexion angle between 120 and 130° could be recommended as an appropriate angle to create the optimal femoral tunnel during ACL reconstruction using the transportal technique.

The present study showed that the knee flexion angle during femoral tunnel drilling had a significant effect on the GBA at the femoral tunnel aperture in ACL reconstruction using the transportal technique. As the knee flexion angle in creating the femoral tunnel increased, the GBA value decreased and the trajectory of the graft at the femoral tunnel aperture tended to become steeper. Because ACL reconstruction has recently been performed using the anatomical reconstruction, the aperture locations of the femoral and tibial tunnels among the elements constituting the GBA are fixed to the native footprints of the ACL^6,30^. Therefore, the only modifiable difference can result from the location of the femoral tunnel exit. According to a previous study on relationship between the flexion angle in creating the femoral tunnel and femoral tunnel exit, the flexion angle significantly influenced the location of the femoral tunnel exit^16,31,32^. The femoral tunnel outlet moved anteriorly and distally with increasing flexion angle. When the outlet of the femoral tunnel moves distally, the value of the GBA (which is the angle formed by the tibial tunnel aperture, femoral tunnel aperture, and femoral tunnel outlet) decreases because the femoral tunnel outlet moves distally toward the tibial tunnel aperture with respect to the midpoint, femoral tunnel aperture. Additionally, because the femoral tunnel aperture is located at the posterior part of the medial wall of the lateral femoral condyle, the graft bending angle becomes more acute when the femoral tunnel outlet moves anteriorly. Considering the change in position of the femoral tunnel outlet according to the change in flexion angle, it can be seen that the GBA becomes more acute when the flexion angle increases. Therefore, an excessively high flexion angle in creating the femoral tunnel should be avoided.

Various factors must be considered to create an optimal femoral tunnel. Femoral tunnel characteristics including femoral tunnel length and the posterior wall breakage are affected by the alteration of knee flexion angle at the time of femoral tunnel creation in ACL reconstruction using the transportal technique^31^. Therefore, to obtain an appropriate flexion angle for femoral tunnel creation, these two factors must be considered. Adequate femoral tunnel length for successful ACL reconstruction remains unclear, however, a femoral tunnel length of > 25 or 30 mm is regarded as sufficient considering the loop length of the cortical suspensory device in the literature^12,33^. In the present study, there were five patients with short femoral tunnel length of < 30 mm and one patient with < 25 mm tunnel length out of 19 patients in group one. All the patients with a knee flexion angle of > 120° had created a femoral tunnel length of > 30 mm. Posterior wall blow-out, which is a violation of the posterior femoral cortex, is a devastating complication that predisposes loss of graft fixation or early graft failure^34^. Lower flexion angle in creating femoral tunnel is related to posterior wall blow-out^35^. In the present study, there was no posterior wall blow-out case. The minimum knee flexion angle for creating the femoral tunnel in the included patients was 112°. Posterior wall blow-out did not occur at knee flexion angles above this angle.

The optimal condition for femoral tunnel creation includes a sufficient length for proper graft healing and incorporation, and a stable tunnel wall without posterior wall blow-out for rigid fixation. In addition to these factors, more obtuse GBA is necessary to achieve better graft healing and graft maturation without tunnel widening^7–10,36^. According to the results of the study, although it is difficult to specify the lower limit of the flexion angle to create a stable tunnel, a knee flexion angle of > 120° might be appropriate to obtain a sufficient tunnel length and minimize the risk of posterior wall blow-out. These results of the present study are consistent with those of previous studies^16,37^. Additionally, an excessively high flexion angle during femoral tunnel drilling can cause more acute GBA leading to unsatisfactory outcomes. To avoid more acute GBA, a lower flexion angle within the possible range is appropriate according to the results of the present study. Considering the various factors mentioned above, knee flexion angle between 120 and 130° during femoral tunnel creation can be recommended for ACL reconstruction using the transportal technique. Most of the existing studies on femoral tunnel of ACL reconstruction have focused on the characteristics of the tunnel itself, such as the tunnel length or the intact tunnel wall. In addition to these factors, a characteristic of the tunnel such as GBA has also been found to affect ACL outcome, and studies on factors affecting GBA values have been conducted. However, what has been revealed is lacking. Moreover, in the ACL reconstruction using the transportal technique, the effect of the knee flexion angle, which has the most important effect on the tunnel creation condition, on the GBA has not been elucidated. In the present study, tunnel creation conditions were obtained considering overall variables including the effect of the knee flexion angle during tunnel creation on GBA. The clinical significance of this study can be found in suggesting an appropriate range of knee flexion angles that can be applied clinically in order to create an optimal femoral tunnel.

There were several limitations in drawing definite conclusions. First, the present study had a retrospective design with a relatively small sample size, which may have contributed to precluding a solid conclusion. To obtain more reliable results, a prospective study with perfect randomization is required. However, the statistical power was calculated to be high (> 80%) for the comparison of GBA. Second, the analysis in this study was performed by comparing three groups. Pearson correlation analysis confirmed that there was a negative correlation between the knee flexion angle during creating the femoral tunnel and graft bending angle. However, a specific cut-off value of the knee flexion angle was not presented because limited data were collected based on clinical data, and all continuous values of the knee flexion angle could not be applied. Third, although the present study revealed the relationship between the knee flexion angle at the time of femoral tunnel creation and femoral tunnel characteristics, their actual effect on the clinical outcomes of ACL reconstruction including femoral tunnel widening or graft maturation was not investigated. It is unknown to what extent GBA changes actually affect the clinical outcomes. Further studies on the long-term clinical outcomes, depending on the variation in GBA are needed. Fourth, the knee flexion angle during creation of femoral tunnel was measured using a sterile goniometer during the operation. Bony landmarks including the greater trochanter, lateral epicondyle of the distal femur, fibular head, and lateral malleolus were used to improve the consistency in the measurement of flexion angle based on previous studies^16^. A single operating surgeon attempted to measure the flexion angle accurately in the same manner, but inaccurate measurement errors may have affected the results.

## Conclusions

Graft bending angle was influenced by knee flexion angle during femoral tunnel drilling for ACL reconstruction using the transportal technique. The GBA tended to be more acute as the knee flexion angle during femoral tunnel drilling increased. By comprehensively considering the length and risk of wall blow-out in the femoral tunnel and the GBA, depending on the knee flexion angle in creating the femoral tunnel, a knee flexion angle between 120 and 130° could be recommended as an appropriate angle to create the optimal femoral tunnel in ACL reconstruction using the transportal technique.

## Data Availability

The datasets generated and analyzed during the current study are not publicly available to protect patients’ personal information, but are available from the corresponding author on reasonable request.
